# Moving survivorship care plans forward: focus on care coordination

**DOI:** 10.1002/cam4.733

**Published:** 2016-04-14

**Authors:** Talya Salz, Shrujal Baxi

**Affiliations:** ^1^ Epidemiology and BiostatisticsMemorial Sloan Kettering Cancer CenterNew YorkNew York; ^2^ Head and Neck Oncology ServiceDepartment of MedicineMemorial Sloan Kettering Cancer CenterNew YorkNew York; ^3^ Department of MedicineWeil Medical College of Cornell UniversityNew YorkNew York

**Keywords:** Cancer survivors, care coordination, clinical trials, health care quality, primary care

## Abstract

After completing treatment for cancer, the coordination of oncology and primary care presents a challenge for cancer survivors. Many survivors need continued oncology follow‐up, and all survivors require primary care. Coordinating the shared care of a cancer survivor, or facilitating an informed handoff from oncology to primary care, is essential for cancer survivors. Survivorship care plans are personalized documents that summarize cancer treatment and outline a plan of recommended ongoing care, with the goal of facilitating the coordination of post‐treatment care. Despite their face validity, five trials have failed to demonstrate the effectiveness of survivorship care plans. We posit that these existing trials have critical shortcomings and do not adequately address whether survivorship care plans improve care coordination. Moving forward, we propose four criteria for future trials of survivorship care plans: focusing on high‐needs survivor populations, tailoring the survivorship care plan to the care setting, facilitating implementation of the survivorship care plan in clinical practice, and selecting appropriate trial outcomes to assess care coordination. When trials meet these criteria, we can finally assess whether survivorship care plans help cancer survivors receive optimal oncology and primary care.

## The promise of survivorship care plans

After completion of cancer treatment, the coordination of ongoing care poses a significant challenge. Cancer‐related concerns remain prevalent. At the same time, cancer survivors continue to have health care needs unrelated to their cancer.

Coordinating the care of cancer‐related issues, the management of general medical care, and the provision of preventive care is critical for quality of life and overall survival in cancer survivors. However, cancer survivors often do not receive primary care 1, 2. A number of studies from the United States have shown that primary care matters. Compared to survivors who visit a primary care provider, survivors who do not see a primary care provider are less likely to receive cancer‐related and preventive services 1, 3, 4, 5, 6, 7. Further, those who see either an oncologist or a primary care provider, but not both, are less likely to receive cancer‐related and non‐cancer‐related follow‐up compared to people who see both types of providers 1, 3, 4, 6, 7. Care coordination is particularly important for survivors with comorbid conditions—cancer survivors are less likely to receive appropriate interventions for comorbid conditions if they see do not see a primary care provider than if they do 8.

While these studies show that primary care is critical, continued oncology care may not be necessary for some low‐risk survivors. A trial by Grunfeld et al. demonstrated that early‐stage breast cancer survivors no longer need to be followed by an oncology team and can safely be cared for exclusively by a primary care provider 9. In this **hand‐off** scenario, coordinated care means assuring that the survivor continues to receive primary care and that the primary care provider is aware of any cancer‐related follow‐up. This may include prevention, early detection, and management of late effects; surveillance for second primary cancers (such as routine mammography); and the process for returning to the oncology team if recurrence or a new cancer is suspected.

In contrast, for survivors who require ongoing care from an oncologist or another member of the oncology team, coordinated care means that both an oncology provider and a primary care provider are involved (in a shared care scenario), and both providers must understand what care is in their purview 10.

In 2005, the Institute of Medicine proposed the use of survivorship care plans (SCPs), which summarize the cancer diagnosis and treatment, describe potential long term and late effects of treatment, and present a plan of ongoing care addressing both cancer‐related and primary care needs 11. Critically, SCPs should include a clear description of which providers are responsible for each aspect of ongoing care. SCPs directly address care coordination and appear well suited to communicate ongoing care recommendations to both the survivor and the primary care provider. However, studies of their usefulness in coordinating care have been inadequate.

## Current state of the science

Although many studies of survivorship care plans exist (see Birken et al. and Salz et al. for reviews), few studies have addressed the impact of survivorship care plans on care coordination 12, 13. Hill‐Kayser et al. provided a survivorship care plan to over 8000 cancer survivors and surveyed them afterwards 14. The authors found that a quarter of survivors shared the survivorship care plan with their primary care provider after a month, and 80% of those who shared the document felt the document had improved communication between themselves and their providers 14. In a quasi‐experimental study of 139 breast cancer survivors, Palmer et al. found that only 21% of survivors shared their survivorship care plan with their primary care provider, but there was an increase in perceived coordination of care after receiving a survivorship care plan 15. Although both studies suggest that SCPs (when used) improve communication with providers, neither study had a control arm, and more importantly, perceived communication may not reflect actual coordinated care between providers.

Ultimately, there have been only four randomized controlled trials of SCPs (resulting in five studies) 12, 13, 16, 17, 18, 19, 20. Each trial has demonstrated little or no benefit 16, 17, 18, 19, 20.

However, it is premature to claim that SCPs are ineffective. A recent editorial by Mayer et al. highlighted that these trials neglect to present information regarding the content and delivery of the SCP—information that is key to understanding whether the null effects are predominantly due to poor implementation 21.

More critically, these trials did not directly address the problem that SCPs are intended to fix: poor care coordination. Handoffs from oncology to primary care must be facilitated, and ongoing shared care provided by multiple providers must be clearly delineated. The Grunfeld et al. trial, with a follow‐up by Boekhout et al., examined a transition in care from an oncologist to a previously identified primary care provider. In the Grunfeld et al. trial, the primary outcome of distress (which was minimal in both trial arms at baseline) was unlikely to be reduced by a facilitated transition to primary care. Similarly, the Boekhout study measured only breast cancer‐specific follow‐up, ignoring the receipt of general preventive care.

In contrast, trials by Hershman et al., Brothers et al., and Nicolaije et al. included survivors who continued to receive care in the oncology setting, in which appropriate care coordination would entail shared care with a primary care provider. It is unclear whether the SCPs in these studies explicitly addressed how the patient would be connected with a primary care provider. Outcomes of the Hershman et al. and Brothers et al. trials were unrelated to care coordination. The Nicolaije et al. trial focused on cancer‐related care and did not measure visits to primary care providers for preventive care, management of comorbidities, or other purposes unrelated to cancer. See Table 1 for a summary of quality‐related attributes of the five existing trials of SCPs.

**Table 1:** Quality‐related attributes of five trials of survivorship care plans

First author (Year)	Care setting	Cancer survivor population	Information in SCP	SCP implementation details	Trial outcomes
Grunfeld et al. (2011)	Complete transfer to primary care	Early stage breast	Treatment summary, follow‐up guidelines, and a resource kit	Delivered in binder in context of nurse visit, data entry for SCP not described	Distress quality of life, patient satisfaction, visits to primary care and oncology providers, and understanding of who provides follow‐up care
Hershman et al. (2013)	Continued oncology care	Early stage breast	*Facing Forward* publication on medical care, potential symptoms, emotions, relationships, and dealing with practical matters (e.g., insurance). Also a treatment summary, surveillance recommendations, discussion of risk for late effects and toxicities, and screening and lifestyle recommendations	Delivered in context of nurse and nutritionist visit, data entry for SCP not described	Treatment satisfaction, concerns about cancer, depression, and the impact of cancer
Brothers et al. (2013)	Continued oncology care	Gynecologic	Diagnosis and treatment summary, late effects of treatments received, cancer screening recommendations, healthy lifestyle information, common psychosocial concerns, and general tips for cancer prevention, among other topics	Created manually and delivered in context of visit with oncologist	Evaluation of quality of care
Boekhout et al. (2015)	Complete transfer to primary care	Early stage breast	Treatment summary, follow‐up guidelines, and a resource kit	Delivered in binder in context of nurse visit, data entry for SCP not described	Recommended and not recommended breast cancer‐specific follow‐up care
Nicolaije et al. (2015)	Continued oncology care	Gynecologic	Diagnosis, treatment, adverse effects	Automatically generated, provided by provider (oncologist, nurse, or both) with suggested discussion topics	Satisfaction with care, satisfaction with information received, concerns, symptoms, emotional impact, and cancer‐related visits to primary care provider

Taken together, these trial findings suggest that in well, low‐risk populations, SCPs may not be effective in improving short‐term health outcomes. Beyond that, there is still much research to do before concluding whether SCPs improve care coordination and, ultimately, long‐term health outcomes. Parry et al. propose a useful conceptual framework in which survivorship care plans are used in their clinical context to influence later outcomes. Specifically, adherence to guidelines, management of late effects and comorbidities, prevention efforts, and health care resource use precede the longer term physiological and psychosocial outcomes. The existing trials often ignore the clinical context, include populations who need less management, and focus on short‐term outcomes 22.

## Recommendations for the development of an evidence base

To evaluate whether SCPs can improve care coordination, trials of SCPs need to be designed carefully toward that end. We propose recommendations for future studies of SCPs.

### High‐needs survivor population

Trials of the effectiveness of SCPs should target high‐needs survivor populations. Survivors with comorbid health conditions, who typically experience serious persistent toxicities of treatment, or who are at high risk of serious late effects may benefit most from care coordination. Coordinating care may help primary care providers understand their role in shared care or sole management of a complex cancer survivor. Although low risk and generally healthy cancer survivors have demonstrated ongoing health concerns, a care coordination intervention may be too blunt a tool to address more minor health issues.

### Tailoring the SCP to the care setting

Each SCP should be geared toward a particular care coordination need. For handoffs to primary care, the SCP should contain information that directs the survivor to seek care from a primary care and informs the primary care provider about the survivor's ongoing needs. Special attention should be paid to informing primary care providers about identifying and managing late effects, as well as any processes for referring survivors back to the oncology team if needed. In contrast, for shared care, the SCP should clearly describe the complementary roles of the oncology and primary care providers, with the goals of getting all appropriate care without duplicating care.

### Facilitating SCP implementation

Barriers to implementation of SCPs are now well‐known. Creating, disseminating, and reviewing SCPs pose a challenge to the implementation of SCPs in clinical practice 23, 24, 25, 26, 27, 28. Future trials should ensure that SCPs are easily created and disseminated—not only in the trial setting, but with careful consideration of potential implementation in future routine clinical care.

The most direct way to simplify the completion of SCPs is to automate data entry. If possible, SCPs should capture patient‐specific information about diagnosis and treatment from electronic sources, either the electronic health record or the cancer registry. As Mayer et al. discussed, data entry and other implementation details must be fully considered and disclosed as part of every trial 21.

### Selecting appropriate trial outcomes

With a long enough time horizon and a large enough study sample, a trial of SCPs may evaluate changes in health outcomes. However, with the null results shown thus far, investigators should focus first on whether SCPs can change the process of care.

Trial outcomes should reflect how well the use of SCPs improves the coordination of care, demonstrated by the successful hand‐off or sharing of care. If the goal is a hand‐off, as was the case in the Grunfeld et al. and Boekhout et al. trials, the assumption is that primary care providers should manage both cancer‐related and preventive care needs. Therefore, trial outcomes should measure the receipt of both types of care. Addressing cancer‐related needs can be measured by referrals to oncology for suspicion of recurrence or new cancers and management of late effects. The receipt of primary care interventions can be measured as the appropriate management of comorbidities, timely screening for other cancers, and vaccination. Trials could also measure differences between study arms in referrals, prescriptions, and other strategies to manage the patients' needs. Primary care providers could report their comfort in being the sole provider of ongoing care.

In contrast, if the goal is to ensure shared care, trials should focus on whether all cancer related and primary care needs are being addressed, whether any are duplicated by different providers, and whether the providers and survivors understand who provides different aspects of care (such as monitoring for recurrence and late effects, treatment of late effects, and screening for other cancers). Trial outcomes could include the survivor's ability to identify a primary care provider, each provider's perception of their responsibilities regarding detection and management of late effects, visits with a primary care provider, and visits with an oncology provider. The receipt of primary care interventions is another way to assess the effectiveness of an SCP in a shared care setting.

All of these outcomes require consistent measurement, but there are few measures that are well suited to evaluating care coordination. Ideally, provision or receipt of care (such as referrals to specialists or receipt of vaccines) should not rely on self‐report, although validated survey measures of care receipt exist. For example, receipt of surveillance testing can be measured with items used to assess screening in surveys such as the Behavioral Risk Factor Surveillance System and the Health Information National Trends Survey 29, 30. Alternatives to self‐report include claims data, registry data, and the abstraction of medical records from both oncology providers and primary care providers. Each of these data sources is limited in some way, such as selective or biased reporting of services in claims data and noninclusion of referrals in a cancer registry. Medical records are likely the most complete and relevant assessments of care coordination, but they rely on consistent recording of care provision and unbiased abstraction techniques.

## Conclusion

SCPs are often promoted as the key to resolving a wide range of challenges after treatment is complete, including emotional distress, lingering toxicities from treatment, and other practical issues that affect daily life. With these expectations, it is no wonder that trials have failed to show benefit. The more realistic promise of SCPs lies in their ability to coordinate care, which has yet to be tested appropriately. The future of SCPs depends on creating a sound evidence base. We hope that our recommendations for the careful design of SCPs and trials will help us understand how these documents can improve care coordination.

## Conflict of Interest

The authors have no actual or potential conflicts of interest to disclose. The authors declare no conflict of interest.
